# Quo Vadis Oncological Hyperthermia (2020)?

**DOI:** 10.3389/fonc.2020.01690

**Published:** 2020-09-04

**Authors:** Sun-Young Lee, Giammaria Fiorentini, Attila Marcell Szasz, Gyula Szigeti, Andras Szasz, Carrie Anne Minnaar

**Affiliations:** ^1^Department of Radiation Oncology, Chonbuk National University Hospital, Jeonbuk, South Korea; ^2^Medical Oncology Department, Ospedale San Salvatore, Pesaro, Italy; ^3^Division of Oncology, Department of Internal Medicine and Oncology, Semmelweis University, Budapest, Hungary; ^4^Innovation Center, Semmelweis University, Budapest, Hungary; ^5^Biotechnics Department, St. Istvan University, Godollo, Hungary; ^6^Department of Radiation Oncology, Wits Donald Gordon Medical Center, Johannesburg, South Africa

**Keywords:** hyperthermia, oncology, trend, immune effects, bystander-effect, abscopal effect

## Abstract

Heating as a medical intervention in cancer treatment is an ancient approach, but effective deep heating techniques are lacking in modern practice. The use of electromagnetic interactions has enabled the development of more reliable local-regional hyperthermia (LRHT) techniques whole-body hyperthermia (WBH) techniques. Contrary to the relatively simple physical-physiological concepts behind hyperthermia, its development was not steady, and it has gone through periods of failures and renewals with mixed views on the benefits of heating seen in the medical community over the decades. In this review we study in detail the various techniques currently available and describe challenges and trends of oncological hyperthermia from a new perspective. Our aim is to describe what we believe to be a new and effective approach to oncologic hyperthermia, and a change in the paradigm of dosing. Physiological limits restrict the application of WBH which has moved toward the mild temperature range, targeting immune support. LRHT does not have a temperature limit in the tumor (which can be burned out in extreme conditions) but a trend has started toward milder temperatures with immune-oriented goals, developing toward immune modulation, and especially toward tumor-specific immune reactions by which LRHT seeks to target the malignancy systemically. The emerging research of bystander and abscopal effects, in both laboratory investigations and clinical applications, has been intensified. Our present review summarizes the methods and results, and discusses the trends of hyperthermia in oncology.

## Introduction

Nowadays, oncology is one of the most interdisciplinary research fields, which includes biology, biophysics, biochemistry, genetics, environmental sciences, epidemiology, immunology, microbiology, pathology, physiology, pharmacology, psychology, virology, and more. Moreover, a wide range of diagnostic and treatment methods are available to identify and destroy malignant tissues. The efficacy of treatment regimens often relies on the fine balance between cure and toxicity and a modality with minimal toxicity and rare complications would be a welcome compliment to the treatment arsenal.

## History of Hyperthermia

Hyperthermia is an ancient physical method and inducing artificial fevers was a common goal for ancient doctors. The development of electromagnetic heating methods in the early 1900s revolutionized the application of heat for therapeutic gains including the treatment of malignancies. In the first quarter of the 19th century, electromagnetism was applied as a healing process (1). The German Electric Belt Agency went further, advertising that practitioners should reduce or even stop using drugs, applying for treatment by electricity alone. A French doctor, Arsene D’Arsonval, introduced a pure electromagnetic treatment called “Darsonvalization.” The absorbed electromagnetic energy resulted in heating, however the effects of heating were initially neglected. Following early observations those increased temperatures in tumors were associated with improved outcomes, and the goal of the electromagnetic therapies shifted to increasing the temperature of the tumors.

The long history of the use of hyperthermia has not benefited the method, and has increased skeptical opposition as various techniques and protocols have been developed over the years which have displayed varying results from positive to negative.

The rapid proliferation of malignant tissue is accompanied by higher biochemical reaction rates (2) and an increase in the tumor’s metabolic rates. This, in turn, generates heat and results in temperatures which are higher than the surrounding healthy baseline temperatures (3). The metabolic heat production of the tumor itself depends on the duration of the doubling of its volume (4). The increase in the chemical reaction rate is demonstrated in the Arrhenius plots which show the reaction change by temperature and this is the basis of the cumulative equivalent minutes (CEM) dosing concept, as described by Urano et al. (5). With a better understanding of the effects of temperatures on tumors, the goal of hyperthermia in oncology shifted to the measurement of the temperature achieved within the treated tumor (6).

The thermal effect of the electromagnetic energy absorption was much simpler to understand and easier to study. The effects of the temperature approach became more robust when Siemens, one of the largest producers of electromagnetic devices at that time, marketed the “diathermia” devices, which were devoted to the cure of cancer (7). Thermo-radiation therapy was approved after a controlled phase II clinical trial with 100 patients in 1912 (8), and further successes were reported by Westermark (9), and Overgaard (10). Intensive publication activity on the development of the methods and their benefits started in the first half of the 20th century (7, 11, 12). A further push on the development was the discovery of microwaves, and a practice working similarly to microwave ovens began. Since then, despite many positive developments and proofs, some uncertainties remain (13).

This positive period for hyperthermia in oncology was followed by one of negative skepticism. In 1964, the leading German surgeon, Bauer, presented doubts about the results in a monograph titled “Das Krebsproblem” (14): “All of these methods impress the patient very much; they do not impress their cancer at all.” His skepticism toward oncological hyperthermia became widespread and common among medical experts, who declared hyperthermia to be of no benefit for cancer patients, and subsequently did not endorse the addition of hyperthermia to therapy protocols. The general policy was to avoid hyperthermia in oncotherapies. Some animal studies even suggested that hyperthermia could increase the dissemination and development of metastases (15–17), and the safety of hyperthermia in humans was questioned (18). The benefits of hyperthermia were however still noted and the investigations began to delve deeper into the methods and protocols (19–26).

A vast amount of research and clinical articles, as well as many books, have demonstrated the efficacy and power of hyperthermia in oncology (27–32). The clinical outcomes are the best ways to determine the treatment efficacy. As hyperthermia is most often applied in end stage cases or to tumors with known resistance to treatments, the most frequently listed primary outcome is local disease control. However more recent studies have included survival and disease free survival as outcomes. In a review of 38 trials on various tumor locations (including breast, cervix, head and neck, rectum, urinary bladder, esophagus, lung, skin melanoma, choroidal melanoma, and anal canal), Datta et al. (33) reports an overall complete response rate (RR) of 54.9% in the 1761 participants treated with hyperthermia plus radiotherapy, compared to 39.8% in the 1717 participants treated with radiotherapy alone. There is a wide variety of techniques available to heat tumors, and each technique requires specific measures of treatment efficiency and safety control. Each technique therefore requires its own evaluation and quality assurance guidelines and these documents are regularly updated as the field evolves (34–38).

Hyperthermia has been introduced within university curriculums (39), and has been discussed in detail in major textbooks on radiology/radiotherapy (40) and general oncology (41). Hyperthermia has now become a part of standard cancer care in some countries, although its application is still limited to only a few cancer types and a few types of heat delivery. Numerous clinical advantages of oncological hyperthermia have been presented (29, 42).

The newest results offer again reason for bright optimism, positioning hyperthermia in oncology (43). Meta-analyses have been published of the most recent medical literature dealing with oncological hyperthermia showing its benefits and perspectives (44–47). A large body of evidence such as this would be sufficient for the inclusion of the more conventionally applied chemotherapy and radiotherapy treatments into routine practice. However, the critical skepticism regarding hyperthermia has not yet vanished. Some of the leading clinical trials have been strongly criticized (48–50). It seems that clear evidence is not enough to convince the experts working in oncology. One renowned expert’s critique formulates an important general consequence: “The mistakes made by the hyperthermia community may serve as lessons, not to be repeated by investigators in other novel fields of cancer treatment” (19). We now ask if the community of radiation oncologists ready for clinical hyperthermia (51).

Despite its long history, the state of oncological hyperthermia today is similar to that of therapies in their infancy. Like many early-stage therapies, it lacks adequate treatment experience and long-range, comprehensive statistics that can help optimize its use for all indications. We are facing the question of what is important for the success of hyperthermia treatments: the temperature or the bio-electric effects, or perhaps both? The search for the explanations of the basic mechanisms of hyperthermia is not yet complete and a new perspective is needed in order to achieve success.

The gold-standard therapies continue to develop intensively: we have seen new radiotherapy techniques (proton and heavy particle therapies, tomotherapy, radiative seeds, etc.), new chemotherapies (antibody therapies, oral medications, check-point inhibitors etc.), and great advances in surgery (minimally invasive surgery, laparoscopic surgery, endoscopic surgery, robotic surgery etc.) in recent years. Unfortunately, oncological hyperthermia has not changed in its thinking regarding the thermal concept that is historically applied. There have been fascinating discoveries in oncotherapies, heading toward immuno-oncology (52); however, the overemphasis on the temperature has remained the leading concept in hyperthermic oncology, ignoring the challenge of the complexity of human medicine (53). In this article we review the technical aspects of various hyperthermia techniques, along with the challenges of the techniques and the advances, and we propose a method of heating tumors which does not require the use of the temperature outcome as a measure of efficiency.

### Hyperthermia Methods in Oncology

A surprisingly large number of hyperthermia methods exist in oncotherapies (54). Different heating processes and technical solutions have been developed (Table 1).

**TABLE 1:** Basic categories of hyperthermia in oncology.

Active physical effect	Example
Heat delivery	Conduction, convection, radiation, bioactive
Energy source	Chemical, biological, mechanical, electromagnetic
Invasivity	Non-invasive, semi-invasive, invasive

## Whole Body Hyperthermia

**FIGURE 1:**
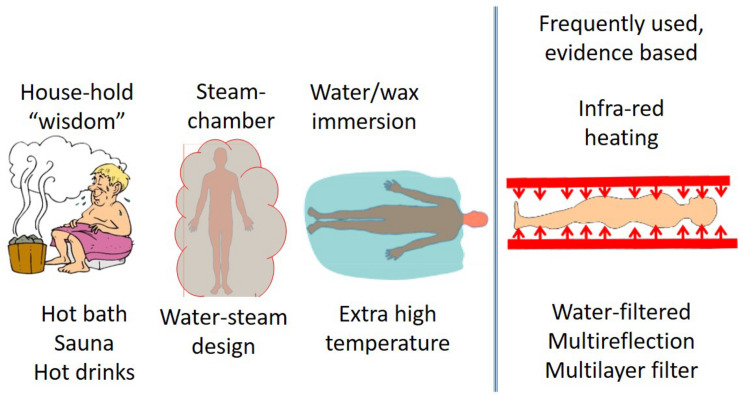
The most frequently used WBH methods. The first three examples do not generate a significant increase in body temperature, however infrared water-window (IR-A) and the extra-corporeal variations of techniques are widely used and have proven to increase the body temperature.

Systemic heating, or whole-body hyperthermia (WBH), is induced by high power technical variants (55), but their common goal is to heat up the blood (mainly peripherally) and for the heated blood to heat up the whole body. Direct contact heating (hot water, hot wax, wrapping the body in a heated blanket etc.) is rarely applied, although water-bath-heating using extremely high temperatures has had its “renaissance” (56). These mainly “heat-conductive methods” have a high risk of burning of the skin’s surface. To avoid burning in the uppermost surface, a so-called water-window effect in the infrared region (denoted by IR-A) of electromagnetic waves is used. This spectrum of the infrared region penetrates deep into the subcutaneous layer and directly heats the blood in the capillaries. Technically this spectrum is selected by multi-reflection filtering (57), water-filtering (58), and multilayer reflection filtering (59). There is also a method by which blood is heated using an extracorporeal setup with outside circulation (60). Whole body hyperthermia methods are illustrated in Figure 1.

## Local and Regional Hyperthermia

Various techniques are used to achieve local-regional hyperthermia (LRHT), all of which have the end goal of the destruction of the tumor. Techniques that have been developed to achieve this include: infrared water-window (IR-A); (61, 62) Hyperthermic Intraperitoneal Chemotherapy (63, 64), [HIPEC: heating the abdominal organs using a warm chemo solution applied during open surgery (65) or laparoscopic surgery (66)] and High-intensity focused ultrasound [HIFU, for deep non-invasive heating (67, 68)]. Photodynamic therapy (PDT) and its different versions are local energy-absorption applications which use the activation of a photosensitizer by the appropriate light dose (usually using a laser-beam in a minimally invasive way) (69). Nanoparticles (70) and even nanotubes (71) are used for modern PDT purposes. The use of laser energy being absorbed by sensitizer-molecules is a form of micro-hyperthermia, which could also be combined with conventional hyperthermia such as interstitial heating (72).

Interstitial heating is an invasive form of local hyperthermia that involves the implantation of arrays of needle-shaped applicators directly into the tumor, or the insertion of applicators within catheters that are implanted into the target volume (37). Non-invasive forms of local hyperthermia include the use of radiofrequency (73, 74) and microwave (75, 76) ablation (RFA and MFA). These techniques assume relatively low energy absorption, but due to the small volume which absorbs this energy, the temperature increases. These emerging technologies are more geared toward minimally invasive surgery and interventional radiology. The ablative necrosis and radical burning solutions differ from gentler hyperthermia effects in that general hyperthermia avoids burning, and describes burns as a toxicity or adverse event [hot-spots and adipose burns (fat-necrosis)].

### The Use of Electromagnetic Energy in Hyperthermia

Energy absorption for heating differs from the ionizing radiation energy delivery method. The used non-ionization electromagnetic effects are sensitive to the bio-electromagnetic heterogeneity of the target volume. The thermal heterogeneity together with the complex feedback mechanisms create challenges as well. The heterogeneity of biological material is complexly regulated by homeostasis. Presently, most of the modern oncotherapies could be combined with hyperthermia (77). Multiple parameters have to be considered in order to optimize the variables of the actual application. The broad range of classifications and technical characteristics of hyperthermia techniques are summarized in Table 2.

**TABLE 2:** Electromagnetic heating methods, based on the heating characters.

Division by electromagnetic heat delivery methods	Technical character
By frequency	Infra, microwave, radiofrequency, low-frequency
By radiation/conduction	Radiative, conductive, mixture
By target distance	Far-field, near-field
By intensity (SAR)	Ablative, active heating, stimulating
By wave-phase	Phase-dependent, independent
By electrolyte conduction	Electrolyte-selective, not selective

**FIGURE 2:**
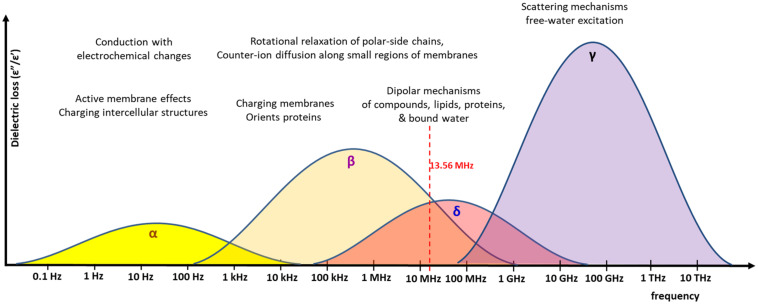
The various frequencies and the associated dispersion. Frequencies have different excitation mechanisms. The energy absorbed at various frequencies (called dielectric loss), is represented graphically for the α, β, δ, and γ absorption range. The 13.56 MHz, which is the carrier frequency of the mEHT, is shown. The distributions are only approximate and depend on the real conditions and heterogeneity of the material, which absorbs the energy. The overlapping of the β/δ ranges has multiple interactions, the effects of which are used for the excitation of membrane rafts, mainly the water-binding transmembrane proteins with their lipid environment.

Frequency dispersion relates to the refraction of the electromagnetic waves as they pass through different mediums. Different tissues in the body have varying biophysical qualities such as protein, water and electrolyte content. In the application of hyperthermia using electromagnetic waves, this interaction between the electromagnetic waves and the medium (the body) is one of the main factors influencing the effects of the treatment. This allows for specific and dynamic interactions between the different layers of tissue, each of which varies in the bio-physical qualities. Figure 2 illustrates the dispersion noted at various frequencies in various tissues.

Dispersion is dependent on the frequency of the energy input. Four dispersion regions have been described at four ranges of frequency: alpha-dispersion; beta-dispersion; delta-dispersion; and gamma-dispersion. The dispersion determines which molecules are affected and subsequently undergo phase transition. In the beta-dispersion range, there is an increase in conductivity of around 0.45 S/m, and an increase of 0.5–0.5 S/m is seen in the delta-dispersion range. Beta-dispersion is primarily due to the capacitive charging of the cell membrane, with a small contribution from the dipolar orientation of tissue proteins. This range therefore effects the cell membranes. Delta-dispersion is primarily due to the broader relaxation of water molecules alone. However, if the effects of the free water molecules are subtracted then the effects of delta-dispersion extend into the radio-frequency (RF) range. Delta-dispersion acts on the lipids and proteins within the cell membranes, as a result of several interactions, including the dipolar relaxation of membrane-bound water, and the rotation and relaxation of the of proteins and polar-side chains. The result is a high absorption of electromagnetic energy by the membrane and protein-bound water molecules and an effect on the transmembrane proteins, and membrane rafts. Modulated electro-hyperthermia, at 13.56 MHz, is between the beta- and delta-dispersion ranges, and therefore makes use of both the effects described above, selecting the transmembrane proteins and the membrane-bound and protein-bound water molecules.

The same forwarded energy exposition with identical energy-flow (W/m^2^) can cause different energy-absorptions depending on the given conditions (78, 79), the actual organ (80), and the actual frequency (81). If the frequency is not correct, the phase transition does not occur and instead the field passes through or around the molecules. In this manner the cell membrane is able to shield the intracellular contents at lower frequencies, but higher frequencies can penetrate the intracellular environment. As molecules absorb the energy, a phase transition may occur. It is therefore possible to target or effect a specific change by choosing the correct frequency input.

The antenna-array coupling is used for energy delivery into the target (82). Its subsequent developments – the annular phase array (83), the matched phase array (84), the Sigma60 (85) and the Sigma-Eye (86) applicators – use a high-frequency RF (60–150 MHz). The antenna array needs a higher frequency which is necessary for the accurate focusing. Unfortunately, these frequencies lie outside the electromagnetic compatibility standards for free frequencies, and therefore require shielding (Faraday-cage). Nevertheless, multiple controlled clinical trials have shown the efficacy of this method (87–89).

An electromagnetic field is an electric and magnetic force field, a property of space, that forms around moving, charged particles. When the charged particles are stationary, causing a difference in the electric potential between two points, voltage is formed between the two points and an electric field forms around the source of the voltage. When the charged particle moves, in other words when there is a flow of current, a magnetic field is also formed, proportionate to the speed of the charged particle and perpendicular to the electric field. An electric field can also be produced by alternating magnetic fields, forcing the movement of charged particles. Some ferromagnetic particles release heat when exposed to an alternating magnetic field. Ferromagnetic particle hyperthermia makes use of magnetic particles, such as micro-particles (90) and ferrite rods (91, 92) introduced into the target volume. Ferromagnetic rods (seeds) have also been used for non-oncological ablative therapies (93). The human body is highly penetrable by a magnetic field. The magnetic field in this application is typically formed between two coils with a high current flow placed on either side of the target volume which contains the magnetic nano-particle. Complications associated with this involve the invasiveness of the introduction of the particles into the target volume (94), however one advantage is the potential reduction in the risk of hot spot formation or damage to the healthy tissues. This method is still considered highly experimental with only a few studies in humans (95). Using the same idea, a new “intracellular hyperthermia” method was developed (96); however, the efficacy of this treatment is still debated (97). The use of nano-particle magnetic suspensions (98) is an emerging magnet-field application. Other types of magnetic heating can be achieved without the inclusion of extra magnetic material into the tumor, and using only the induction of Eddy-currents (99–101). Although the magnetic field goes through the body, the electric field does not, and the method has a low heating efficacy. Due to problems in the selective targeting of the tumor this method is less popular.

Applications making use of capacitive and the radiative (microwave) solutions have the highest popularity in technical realization; however due to the sharp decrease of penetration depth with the increase of frequency, microwave solutions are mostly applied for surface lesions. The capacitive coupling of energy delivery has become the most frequently applied technique. This coupling has two categories, depending on the matching to patients: plane-wave radiation and resonant impedance matching (favoring the highest available RF-current).

Capacitive coupling has less contraindications than other electromagnetic solutions, due to its simple arrangement, and so sensitive tumors such as those of the lung and the brain can be treated by this technique. The efficacy of capacitive coupling has been verified and validated in the relevant literature (102–104). The RF for the capacitive solution is typically in the 5–30 MHz radiofrequency range, and the preference is mostly for the so called “free-frequencies” (13.56 MHz, as well as at half and double that frequency). These free frequencies are approved for industrial, scientific and medical use (ISM frequencies) (105).

Modulated electro-hyperthermia applies capacitive coupling with impedance matching in order to maximize the absorbed energy and minimize the reflected, or lost energy (106). Due to the high efficacy of current matching (107), the absorbed energy can be used as the dose control (108, 109) instead of the temperature achieved within the tumor.

**FIGURE 3:**
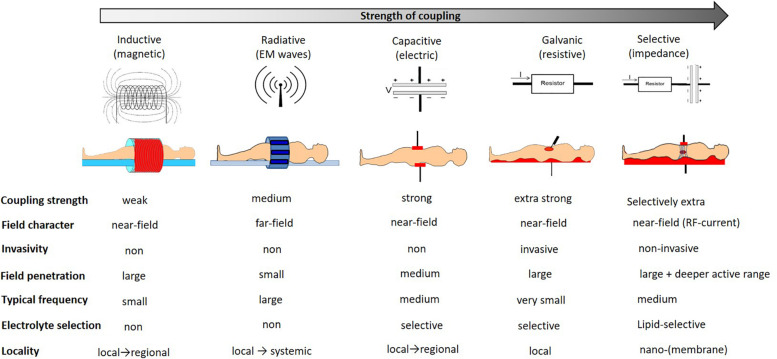
The interaction strengths of the bio-electromagnetic interactions. The “strength” refers to the ability of the energy to be absorbed by the living system.

Various bio-electromagnetic interactions occur based on the frequency of the field and the coupling strength of the fields to the target (Figure 3). Galvanic coupling typically provides the strongest (most effective for energy delivery) interaction. However applying other coupling methods, such as capacitive coupling, with impedance matching could improve the efficacy by minimizing energy losses and enhancing resonant energy-absorption, thus exceeding the strength of the galvanic coupling.

**FIGURE 4:**
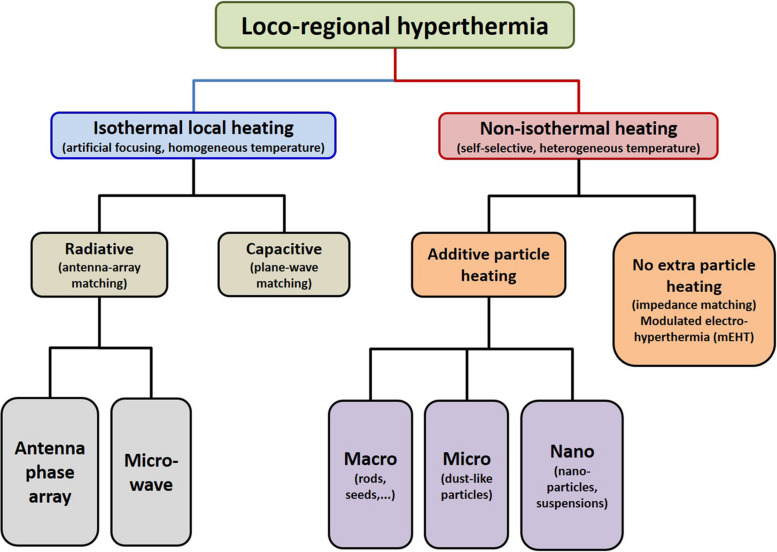
Basic division of loco-regional hyperthermia categories (112). There are technical solutions in each categories, and each product has its own unique technical details, but all have the end goal to heat, either resulting in either temperature homogeneity or heterogeneity.

Electromagnetic loco-regional hyperthermia is a large branch including various methods, but two basic principles are distinguishable: isothermal and non-isothermal heating (Figure 4). The isothermal approach aims to heat up the tumor equally, as homogeneously as possible. Two basic technical solutions are used for isothermal (homogeneous) heating: the radiative and the capacitive methods, as depicted in Figure 4. Non-isothermal hyperthermia heats up the tumor non-homogeneously, by selecting characteristics unique to the target volume. The inhomogeneous characteristics of the tumor are heated, but the temperature of the tumor as a whole is not necessarily evenly distributed. Two techniques are applied to achieve non-isothermal heating: particle heating, which uses particles injected into the target volume; and modulated electro-hyperthermia (mEHT) (110), which exploits the selective absorption of energy by glycolipoprotein lipid micro-domains (membrane rafts) (31).

Microwave heating revolutionized local hyperthermia in the 1950s, when the promise of deep heating by focusing on the tumor inside the body was highly attractive and ignited numerous projects. While the dose of the microwave heating in the oven is measured by the applied power and duration of the radiation, hyperthermia for humans could not follow this measure. Isothermal heating involves trying to achieve the best temperature homogeneity of the target. The heating efficacy is uncontrollable in the medical applications due to two reasons:

1. The physiological effects control the thermal homeostasis, the transport of the electrolytes (like blood, lymph, extracellular matrix) appear as a heat-sink of the absorbed energy and create massive thermal inhomogeneity in the non-homogeneous tumor-mass.
2. The presently available techniques are insufficient for focused isothermal deep heating. The variations of the thermal qualities within the targeted volume are non-linearly enhanced by the absorbed energy.

Regardless of the difficulties, the absorbed energy in the targeted body part is estimated based on the achieved temperature, and temperature became the basis of the dose-determination. Furthermore, due to the synergy between hyperthermia and other highly temperature dependent treatment techniques, the primary focus was to enhance the temperature.

Nevertheless, the measurability of the achieved temperature does not consider the thermal heterogeneity of the target. The dose predicts the percentage of isothermal volumes of the target. However, the enormous micro-heterogeneity of the tumor mass due to the intensive and non-linearly regulated blood flow does not allow the real mapping of the temperature in the targeted volume.

## Effects of Hyperthermia

The effects of increased temperatures on cells have been described in detail in the literature over the last four decades (33, 111–114). Despite the plethora of pre-clinical research, the exact mechanisms of some of the effects of hyperthermia remains uncertain. *In vitro*, temperatures in excess of 41°C inhibit the DNA double strand break repair mechanisms by slowing down the synthesis and function of proteins. The dependence of this action on temperature *in vivo* is however not yet clear.

Mild temperatures have demonstrated immune-modulating potential and the induction of systemic anti-tumor immune responses (115). Local irradiation combined with combined mild hyperthermia may result in systemic effects through immune mediated abscopal effects (116–118). Temperatures in excess of 43°C can result in the direct destruction of cells, via necrosis, rather that the synergistic action milder temperature’s with chemo-/radiotherapy. Necrosis at these temperatures is primarily achieved by the denaturation of proteins, which are essential to the maintenance of a healthy cell cycle.

Hyperthermia has the added effect of sensitizing cells which are in the S-phase of cell division, a phase during which cells are more likely to be resistant to conventional therapies (33). Hyperthermia with bio-electromagnetic selection targets the tumor and complements the effects of chemo- and radiotherapies due to the chemo-action being mainly in the S-phase of the cell’s cycle (119), while the ionizing radiation is most active in the G1 and mitotic (M) phases. For chemotherapy this support is effective when the chemo-action misses the S-phase [as with Mitomycin (acts in the G1 phase), taxan-derivatives (act in the M-phase) or Epirubicin (acts in the G2 phase)].

The robust synergy between chemotherapy and heat is attributed to the thermally increased metabolism (enhanced chemo-metabolism) and the increased absorption of cytotoxins (120, 121) and cellular chemo-penetration which is promoted by non-equilibrium heat-flows (electro-osmosis) (122). An increase in temperature accelerates the pharmacokinetics (improves the reaction rates). This effect could also be complemented by the temperature sensing of the actual drug (5). Thermo-chemotherapy results in a more efficient therapeutic effect increasing the target specificity and reducing the systemic side effects (123). In some cases, lower-dose chemotherapy could be used (124–126) with hyperthermic promotion. Such optimized chemo-intake helps to overcome the failing of chemotherapies due to patient intolerance (when they are not allowed to take large doses of drugs – for example, due to renal or liver insufficiency or insufficient blood composition). In these cases, the same results may be achieved by combining a decreased chemo dose and heat-therapy (127).

Other advantages of increased temperature include alterations in the cellular membrane, by softening or melting the lipid bilayer (128, 129), and changes in lipid-protein interactions (130). Heat-treatment causes a structural alteration in transmembrane proteins, causing a change in active membrane transport and membrane capacity (131), leading to substantial changes in potassium, calcium, and sodium ion gradients (132), membrane potential (133, 134), and cellular function (135, 136). Hyperthermia increases biochemical reaction rates and, therefore, the metabolic rate. The anaerobic metabolism quickly impoverishes the ATP sources and produces lactate (137), causing hypoxia. The ATP depletion increases ionic imbalance in cells further (138). Another benefit to hyperthermia treatments is the management of pain (139).

### Perfusion

The major factor in homeostatic control is blood circulation. Hence, as a consequence of the increased temperature, multiple factors need to be modified to make this addition to cancer treatment a success. Temperature increases of +2°C above the normal range result in vasodilation (140, 141), while temperatures in excess of 43°C result in vasoconstriction (140). Vasodilation promotes blood and oxygen perfusion which in turn increases the delivery of chemotherapy and enhances the cell killing effects of chemotherapy and ionizing radiation. The increased cell killing effects from ionizing radiation are primarily (but not exclusively) due to an increase in the PO2 in tumors (142), and the increased cell killing effects of chemotherapy is due to a combination of increased drug delivery, increased metabolic rates of the heated cells, and the synergistic effects with the various actions of the drugs on the cell cycle.

The most active regions of a tumor and regions far from the blood supply are usually severely hypoxic; therefore, radiation has reduced efficacy in these areas. The possible vasodilatation caused by hyperthermia aids the synergy by increasing the overall blood perfusion (oxygenation) (143), creating a considerable sensitization to ionizing radiation. This approach was one of the first of the modern hyperthermic effects to be studied, and its characterization was introduced by the thermal enhancement ratio (TER) (144), measuring the efficacy of the treatment. Improving the efficacy of radiotherapy may allow for the possibility to apply a reduced therapeutic dose of radiotherapy, promoted by hyperthermia using the complex TER factor (145, 146). Various review articles have summarized the sensitizing of the classical ionizing radiation by hyperthermia (145, 147–150).

The vascular response to the growing temperature, and the effect of this response on the heating of the tumor, is a complex phenomenon. It has been shown that an increase in temperature can cause vasocontraction in certain tumors leading to decreased blood perfusion and heat conduction (151–153) in the neo-vascularized area. The tumor’s blood flow depends on the tumor’s weight as a negative logarithmic function (140, 154), which is a further modifying factor dependent on the development of the tumor’s mass; a more developed tumor has less blood flow because of vasocontraction. So, the blood perfusion of a larger tumor relative to the surrounding healthy tissue is always lower (155) providing an effective heat trap (156, 157), due to the epithelium of new vessels differing from the normal vessels (158). In small tumors and also in the surrounding healthy tissues, the temperature growth causes vasodilatation, which increases the heat conduction in this region (159, 160). The development of blood flow differs in healthy and malignant areas in small tumors too (147).

The mechanisms of physiological regulation and the role of blood flow was not fully understood during the times when hyperthermia in oncology was being developed. The homeostatic regulation devoted to maintaining thermal homeostasis induces a vascular response (140, 154) which increases the blood flow in the heated area, where the fresh blood is a cooling medium. The heated local target is cooled by the blood non-linearly. While the thermal homeostatic control tries to cool down the heated volume, the healthy tissues show vasodilatation. The increased blood flow delivers not only more drug and oxygen, but also supports the tumor-growth with extra nutrients – mainly glucose. This initiates an uncontrolled competition between the thermal damage and the transport support. Furthermore the healthy region induces higher flow than the tumorous (159), and the flow-gradient supports the invasion and dissemination of the malignant cells, which increases the risk of metastases (161). Increased blood flow may therefore pose a risk by supporting blood-delivered glucose and other nutrients and potentially aiding in the dissemination of malignant cells, forming micro, and later macro, metastases.

### Challenging the Isothermal Heating

**FIGURE 5:**
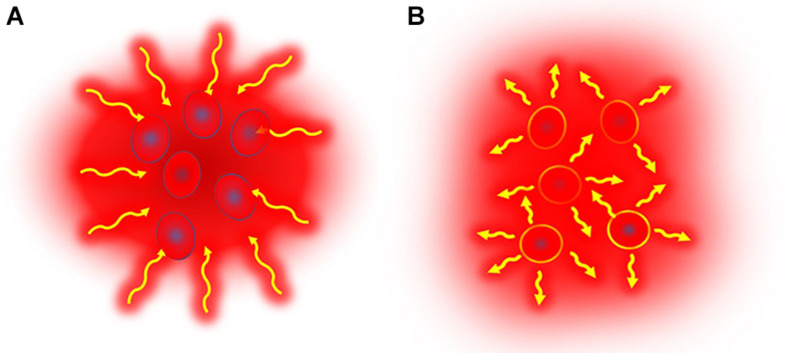
Illustrative representation of the two heating principles. **(A)** Isothermal (homogeneous) heating causes energy-absorption in the complete target, while in **(B)** non-isothermal heating only selected parts are heated in a heterogenic manner. The selected parts that are heated are either unique characteristics of the tumor which respond to a stimulation by heating up, or nano-particles which are introduced into the tumor and which respond to a stimulus by heating up. These heated targets then heat up the target volume by heat conduction.

The dose considerations concentrate on the percentage of the volume which could be considered as having isothermal status. Complete homogeneity of heating of living objects could be achieved only in the WBH process, as LRHT has huge anatomical, physiological, bio-electromagnetic, and thermal heterogeneities, which limits the isodose-type approach. Heterogenic heating with microscopic (cellular) selection does not have such a limit. The applied electromagnetic field targets particles and these particles can be supposed to have equal absorbed energy-doses, so here the absorbed energy is the measured parameter. The particles in this method may be foreign magnetic particles introduced into the target volume, or particles which already exist within the tumor, The homogeneous heating method heats all parts of the target from outside, while the heterogenic heating heats only the selected particles and those heat up the tumor where they are located (Figure 5). The selected particles are heated up intensively to have a higher temperature than their environment. To obtain thermal equilibrium the heat moves naturally from the heated target particles to the environment and in doing so heats up the complete target.

The conventional homogeneous heating process has been the subject of a number of reviews. The most recent books about the physics (30) and biology of the process, from research to clinical use (162), show detailed and comprehensive insights into the topic. The use of nanoparticle heating is heterogenic in that the target volume is not heated equally but rather only the targeted particles are heated. However the outcome is a homogenous distribution of the heat as the system moves to re-establish thermal equilibrium. Considering only the outcome and not the method, some reports continue to describe nanoparticle heating as homogenous.

**FIGURE 6:**
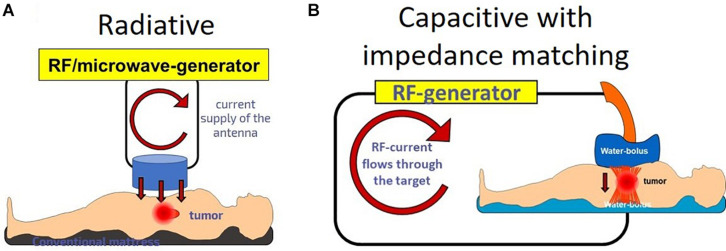
Radiative and capacitive heating with plane-waves. **(A)** The radiative situation causes an artificial focusing. The target is independent from the source. **(B)** Capacitive with impedance matching: the source and the target are coupled; they are in a common electric circuit.

The homogenous principle is applied in radiotherapy treatment planning when the target volume for delivery of conformal dose of radiation is delineated based on the previously measured patient data. Treatment planning is completed independently of the patient’s actual status, movements, changes, and demands at the precise time of irradiation. Sophisticated techniques have been developed to adapt the treatment to the margins as they change with fine movements, such as breathing, during the treatment and with the subtle fluctuations in the patient’s composition between treatment fractions. In homogenous hyperthermia, the development of equally sophisticated techniques to delineate margins and spare the surrounding healthy tissues are necessary. Applying a heterogenic principle however, the need to delineate margins and to treat the complete tumor is reduced, as only the targeted nano-particles (introduced or naturally occurring), are treated. This enables a self-focusing and continuously adaptive treatment. The target forms part of a resonant electrical circuit which *in situ* and in real time retunes to any changes or movements, keeping the full system in a controlled synchrony, as illustrated in Figure 6.

### Efforts to Reduce the Energy-Loss

**FIGURE 7:**
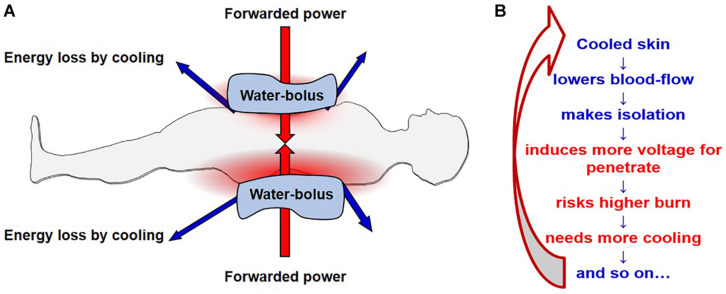
The challenge of electrode cooling. The figure shows the capacitive solution, but the situation is the same with the radiative solution, only with three-dimensional cooling (water bolus typically wraps around the patient) instead of in the two dimensions of the parallel pads. **(A)** The forwarded energy dose is compromised by the energy removed by cooling mechanisms. **(B)** The situation starts a physiological reaction which results in a positive feedback cycle: the cooling causes are reduction in blood flow to the skin, causes electrical isolation in the superficial tissues, requiring an increase in the power (voltage). This in turn increases the burn risk, so more cooling is needed, further decreasing the blood flow. And the cycle continues in a positive, non-linear loop.

While the WBH technology uses the absorbed energy in the body and tries to keep it in the body without losses, the LRHT methods work differently. To heat up a deeply situated target, which is cooled down by the intensified blood flow in a controlled non-linear physiologic loop, LRHT needs a high intensity energy flux on the surface over the target. All the LRHT coupling methods need surface cooling to avoid the overheating of the skin causing thermal toxicity such as epidermal or subcutaneous burns. This cooling helps improve the safety of these methods, but causes two consequences which require consideration (Figure 7):

• The uncontrolled energy which is taken by the cooling process will be missing from the amount supplied. Due to the lost portion of the energy being unknown, the overall energy supplied cannot be used as a treatment dose parameter, the energy balance of the penetration loses its trustworthiness.
• The physiological regulation of blood flow makes the situation complicated (163). The cooled surface will experience vasocontraction, and this layer will become more insulative, as a normal physiological reaction, limiting heat loss. This extra isolation means a higher voltage becomes neccessary to push through the electromagnetic energy, which decreases the current in the fixed power conditions. The higher voltage has more risk of causing surface burns of the electrical kind, so the surface needs more cooling and the process enters a positive feedback loop. The cooling destabilizes the dynamic equilibrium, decreasing the electric current intensity, which is the main factor upon which the expected heating effect depends.

**FIGURE 8:**
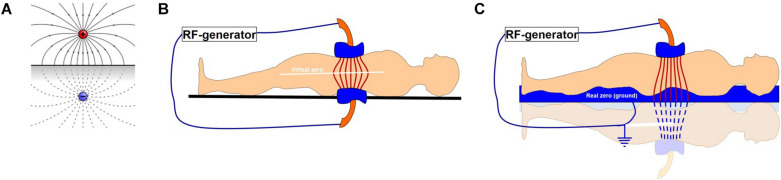
A representation of the effects of two electrodes with opposite charges. **(A)** Two opposite charges, **(B)** two plan-parallel electrodes in a capacitive coupling forming a virtual zero field in the center between each electrode, **(C)** a one-side grounded capacitor, with one electrode and the bed base acting as the second, grounded capacitor, as applied in the impedance matched coupling technique.

The capacitive coupling methods (including impedance coupled mEHT) have the further challenge of the distribution of the electric field in the body. It is a crucial point, as the electric field generates the necessary heat-production and the excitement of the molecules by their absorption of energy. Alternating the charge of two symmetrical applicators (electrodes) between positive and negative causes the plane in the middle of the plan-parallel electrodes to have a zero potential. This is due to the canceling effect of the two opposite fields generated by the opposite potentials of the electrode-plates. When one of the electrodes is larger, the symmetry is disturbed and the zero potential plane shifts toward the smaller electrode. When one of the electrodes is grounded, a zero field is created at the point of the grounded electrode, and the field is mirrored, which we can treat as if it were two mirrored patients with one of the electrodes grounded (Figure 8). Both of these solutions have particularities.

The electric potential is zero at the midway point between two equal but opposite charges. Alternating two identical (symmetrical) but oppositely charged electrodes will create the same effect: a plane of zero potential midway between the electrodes. This “virtual zero” point between the symmetrically alternating potentials on the electrodes is very sensitive to the actual symmetry of the target as well as the symmetry of the electrodes. In this technique the tumor should not be in the center, but should be closer to one of the electrodes (ideally the small electrode). This symmetry is not only geometrical but is also considered as symmetrical in its distribution throughout the body, regardless of the non-homogenous quality of the body. The non-homogenous biophysical characteristics of various tissues within the body (for example the bone, adipose tissue and muscles), cause a variation in the response of various tissues to the field. The variations in the biophysical properties (such as conductivity and impedance) are caused by variations in the concentrations of proteins, electrolytes, and water, as well as other factors. The body itself is not symmetrical and the field is therefore disturbed by the variations in the biophysical properties of the tissues through which it must pass. The virtual zero also moves according to outside disturbances (nearby medical staff, near zero potential environmental parts like walls, metal furniture etc.). This makes it difficult to control the field in order to ensure maximum effect on the tumor. This challenge is well known and described elsewhere in the literature. When one side is grounded however, the entire patient falls into the field and the depth of the tumor is less important (107).

### Complementary Combinations of Hyperthermia With Other Therapies

A large number of publications have described the synergy between conventional ionizing radiation therapy and hyperthermia (148–150, 164–167). The multinational Radiation Therapy Oncology Group (RTOG) also evaluated the method as feasible (168). Due to the sensitization, a reduced dose of radiotherapy could be applied in combination with hyperthermia (145, 147, 169). Hyperthermia supports the application of complementary chemotherapies by the increased chemical reaction rate, which grows exponentially with the temperature (109, 170–172). The variety of thermo-chemotherapy and thermo-radio-chemotherapy combinations available offers greater local control of the tumor with less side effects.

The temperature sensitivity of the drugs applied supports the complementary application of hyperthermia with chemotherapies (5). Thermo-chemotherapy results in a better therapeutic effect and increases the target specificity as well as reducing the systemic side effects (89, 124). In some cases hyperthermia may improve the efficacy of low-dose chemotherapy (125, 127).

The most well-known phase III randomized controlled trials demonstrating improved local control and survival, were in the head and neck (173), breast (47), cervix (174, 175), sarcomas (176), and melanomas. A clinical review is outside of the scope of this article, however Datta et al. provides an excellent summary of the clinical outcomes of the phase II and III hyperthermia trials which had been published by 2015. In the five years since the review, a phase III randomized controlled trial on mEHT with/without chemoradiotherapy in HIV positive and negative participants has shown improved outcomes (177), and a randomized controlled trial on bone metastases treated with radiotherapy with/without hyperthermia showed a significant reduction in pain and improved palliation (178). A ten year survival report of sarcoma patients treated with chemotherapy with/without hyperthermia showed significantly improved survival rates (179). Two retrospective studies, one of which was a multicenter, double armed study, showed an improvement in survival and disease stabilization of recurrent and resistant brain (180) and pancreatic tumors (181) treated with mEHT versus best supportive care.

#### Cervix

One of the most investigated localizations of hyperthermia therapy is for the uterine cervix. Its combination with chemotherapy (Cisplatin + hyperthermia for previously radiated cases) also shows feasibility (182, 183), as well as the combination with radiation (184–186) showing great success. However new evidence (176) fueled the debate with counterpoints (187), counterarguments (188, 189), further debates (190), and responses thereto (191). Some further bimodal (192, 193) and trimodal applications for the cervix (194–196) were also reported regarding locally advanced disease. Conventional and network meta-analyses were conducted for locally advanced evidence-based clinical results which showed the definite efficacy of hyperthermia (46, 48). The outcomes of radiation therapy with radiative hyperthermia or with chemotherapy are comparable (197). The mEHT method was effective in advanced cervical tumors (184), and it has been shown in a Phase III trial that the benefits extend to distant metastases when cervix tumors are treated locally with mEHT and radiotherapy (198).

#### Brain

Such sensitive tumors as gliomas are complicated to treat with hyperthermia. The increase of the brain temperature could increase the intracranial edema and intracranial pressure, which could be fatal. However, due to the absence of effective traditional therapies, a relatively high interest in studying heat-effects on the brain is present (199, 200). Due to the risk of increasing intracranial pressure, the precise localization of the incident energy and the precise selection is essential. Numerous, very local, invasive (ablative, interstitial) hyperthermia treatments combined with local irradiation (201–210), in combination with laser techniques (211, 212), implant applications (174, 213), and nano-particle magnetic heating (214) have been applied for the treatment of gliomas. A postoperative application has also been published (215). The combination of interstitial hyperthermia with external radiation has also been tried (216). One of these, interstitial (minimally invasive) hyperthermia, was applied in a randomized, controlled double armed (with and without hyperthermia) clinical study (217). It showed a surprisingly good efficacy for brain-gliomas: the median survival period grew from 76 to 85 weeks, and the 2-year survival period was up to 31 from 15%. Subsequently the FDA certified brain-interstitial hyperthermia. Some radiofrequency hyperthermia techniques have been applied intra- and extra-cranially (175, 218, 219), as well as ultrasound hyperthermia (220). It has also been shown that the electric capacitive coupling (called Electric Capacitive Transference) could be effective when applied transcranially (221). The non-invasive electric field application (tumor-treating fields, TTF), without an observable increase of temperature, had remarkably good results, proven by Phase III clinical trial (222), and was approved by the FDA. Modulated electro-hyperthermia has also been applied for gliomas without notable side effects (177, 223–226). Immuno-addition increases the efficacy of mEHT (227), and the economics are also better in this application (228).

#### Pancreas

A vast proportion of pancreatic tumors are inoperable and only a few options are available for curative treatments. One of the earliest extended therapies studied for the pancreas (229) was with *n* = 77 patients with resectable and non-resectable adenocarcinoma. The method was capacitive coupling at 13.56 MHz as an immune stimulator. The patients who received hyperthermia got a lower chemotherapy dose. The two groups were compared: with and without the addition of selective immune stimulation. The first-year survival percentages and the median survival period are collected in Table 3. For comparison, three studies of chemotherapies without hyperthermia (230–232) gave 25, 22, and 28% 1-year survival ratios and 6, 7, and 6.5 months median survival periods, respectively.

**TABLE 3:** First-year survival percentages and MST of pancreas hyperthermia treatments (capacitive, 13.56 MHz).

Additive therapy	AII	Far-advanced diseased (%)	No response on conventional (%)	Operated (bypass or resected) (%)	Median survival time (MST) (m)
Immune stimulation	35	13.3	34.1	48.7	8
No immune stimulation	6	0	0	11.1	6

The minimally invasive ablative hyperthermia technique has also been successfully applied for unresectable pancreatic carcinomas (233), and the feasibility of the treatment of pancreatic cancer with hyperthermia is shown by a meta-analysis (234). Successful clinical trials have shown the advantages of the mEHT method (235–237), applicable also when conventional therapies fail (238).

#### Breast

Breast cancer is also frequently and successfully treated with hyperthermia in combination with radiotherapy, showing significant advantages compared to radiotherapy alone (239–241). Results of five controlled clinical trials were collected showing the feasibility of hyperthermia in chest wall recurrences in breast cancer (242). Capacitive coupled hyperthermia in combination with radiotherapy has been compared to radiotherapy alone (243) in recurrent and advanced cases (244). It is shown (245) that when the tumor is larger, the local response is better, the gain in the efficacy compared to radiotherapy alone is 13.7% when the tumor is smaller than 100 cm^3^, and 22.6% when it is larger than 100 cm^3^. The 4-years overall gain in survival period is almost four times higher (246) with chemotherapy (liposomal doxorubicin) (246), and with trimodal therapy (247). A benefit has also been observed in non-inflammatory cases (248). The ablation technique has also been applied with success (249). A meta-analysis was performed (45) to show the present status of hyperthermia in the therapy of breast cancer. The mEHT method is also applied with success in various advanced cases (250–252), and a randomized clinical trial is expected for complete proof.

#### Lung

Some successful clinical trials in combination with radiotherapy have shown the feasibility of the hyperthermia method for non-small-cell lung carcinoma (NSCLC). Most of these are combined with radiotherapy, having 14–70 Gy dose in the given cycle. The measured RR was surprisingly high RR = 75%, [*n* = 12 (253)], and RR = 100% [*n* = 13 (180)].

A study of advanced NSCLC patients (*n* = 13, capacitive coupling, *f* = 8 MHz) to control the local chest invasion (254) showed similarly good results, and the pain-relief was also surprisingly good. Locally advanced NSCLC patients were studied (*n* = 32) with fractional radiation (180–300 cGy/fraction, 5 fraction/week, median dose 5.58 Gy) (255). Results indicate differences, but they were not significant. The 13.5 month median survival period of the historical control was increased by postoperative (lobectomy or pneumonectomy) application of intrathoracic chemo-thermotherapy (bolus intrathoracic infusion of cisplatin followed immediately by hyperthermia), to 17.5 months, by capacitive coupled hyperthermia (256, 257). The postoperative application of hyperthermia has also been successful in other studies (258). The results of another clinical study (*n* = 80) on NSCLC shows no significant differences between the active (hyperthermia plus radiotherapy) and control (radiotherapy alone) arms (259) in the local RR but the local progression-free survival period was significantly better (*p* = 0.036) in the hyperthermia arm, although the number of metastases also increased.

The combination with chemo-thermotherapy has also been investigated for NSCLC with success. In preclinical trials Cisplatin was shown to be effective (260), so clinical studies have concentrated on this drug and its combinations. The synergy between Gemcitabine and hyperthermia in NSCLC was shown *in vitro*, and *in vivo* on nude-mice xenograft model (261). The decrease of the tumor-size and a significant inhibitory effect on growth were shown, and the hyperthermia support of the Gemcitabine induced apoptosis was also demonstrated.

Whole-body hyperthermia has been applied for advanced lung cancer (262). This study (*n* = 49) showed an effective benefit of hyperthermia, which was more effective in elderly (>60 year) patients. The remission rate was 50%, and the median survival time (MST) was 7 months (mean was 12.7 months) in primary and 5.5 months for metastatic diseases.

Percutaneous ablation with radiofrequency (181, 263) and with laser induced interstitial thermotherapy (264), are also in use for pulmonary tumors. The intrapleural hyperthermia by perfusion is also in use in clinical practice (265). A more experimental technique that appears feasible involves filling the lungs with non-toxic, breathable perfluorochemical (PFC), and subsequently heating the region (266).

The mEHT treatment has successful clinical records on lung cancer (267), and case reports of advanced and special cases support its application (268, 269). When mEHT was applied to lung tumors simultaneously with intravenous ascorbic acid the plasma concentration of ascorbic acid was significantly increased, compared to intravenous ascorbic acid applied alone, or after mEHT. This suggests that mEHT enhances the pharmacokinetic parameters as well (270). An important development is the good results on small-cell lung cancer (SCLC) (271), which opens new perspectives for mEHT in this field.

#### Liver

The liver is one of the most problematic organs for cancer, because of the poor prognosis of primary hepatocellular carcinoma (HCC) and the frequent liver metastases from a large variety of other localizations. The liver can be successfully treated by local chemotherapy (chemoembolization), which is one of the most popular and successful chemo treatments. Hyperthermia is an excellent synergetic completion of chemoembolization, increasing the remission rate by more than 12% (272). The result is remarkable for larger size tumors. Others have supported these results (273, 274). Hyperthermia works in synergy with numerous different therapies, and all have achieved good results in HCC and also in non-HCC studies (275). Hyperthermia with chemoembolization has been successfully applied for melanoma metastases in the liver (276). Other studies (277–279), have shown excellent complementary results for metastatic liver tumors. Hyperthermia is successfully applied in combination with radiotherapy for non-resectable cases (280), and various ablation techniques have been applied to eliminate liver tumors. Ablation can be done by laser (281), laser in combination with chemoembolization (282) or with the RF ablation technique (283). The application of mEHT has been investigated for the treatment of liver metastases from colorectal cancer and the median survival period in the patients treated with mEHT, with or without chemotherapy (5-FU + FA + MMC) of colorectal metastasis more than doubled (284, 285) and the disease was stabilized (286).

#### Colorectal

In a study on radiotherapy combined with capacitive hyperthermia for recurrent or non-resectable colorectal tumors only two cases showed progressive disease (287). Similar results were obtained in other studies (288–290) as well. Success could be obtained by applying hyperthermia together with chemotherapy in the case of pre-irradiated lesions (291). Preoperative hyperthermia applications were also successful in the trimodal (chemotherapy, radiotherapy, and hyperthermia combination) approach (292–294), and even intraoperatively (295). Peritoneal carcinomatosis has also been treated successfully with hyperthermia (296, 297).

#### Esophageal

Good results are obtained for esophageal carcinoma treatment by capacitively coupled (intraluminal 13.56 MHz) hyperthermia (298, 299). A histopathology examination revealed the treatment effect of each type of preoperative adjuvant therapy. The effective rate was 68.8% in the hyperthermo-chemoradiotherapy (HCR) group and 44.1% in the chemoradiotherapy (CR) group (*p* < 0.05). The survival rates were 50.4% in the HCR group and 24.2% in the CR group. Results were shown in comparison with other studies (300, 301). The treatment efficacy shows a difference in comparison with and without hyperthermia, and could also feasibly be applied preoperatively (302). Results have shown benefits to the use of hyperthermia in combination with radiotherapy for the treatment of recurrent esophagus carcinoma (303) and also when combined with chemo-radio-therapy (299, 304–307).

#### Head and Neck

The results of capacitively coupled hyperthermia in head and neck carcinoma have also shown definite advantages (308). Curative resection after locally applied radiotherapy with hyperthermia is also feasible (309). An important result for radiotherapy combined with hyperthermia is the observation that the hyperthermia synergy is much higher in the advanced stages than in early-stage cases (310). A randomized study (311) and a summary of clinical studies (312) show improved outcomes when radiotherapy is combined with hyperthermia, including improved 5 year survival rates (313). Improved outcomes have also been demonstrated with the addition of hyperthermia to chemoradiotherapy (cisplatin) (314). A meta-analysis of six clinical trials including 452 participants reported an odds ratio of 2.92 (95% CI: 1.58–5.42, *p* = 0.001) for local control in favor of hyperthermia combined with radiotherapy versus radiotherapy alone (178).

#### Gastric

The efficacy of intraperitoneal chemo-hyperthermia for gastric cancer patients with peritoneal carcinomatosis was better for the hyperthermia group, but the results were not significant (315). The radiotherapy combined treatments have been effective in most of the trials (316). Both preoperative (317) and postoperative (318) treatments have been successfully applied, along with hyperthermia combined with radiotherapy (318), chemotherapy (317, 319), and chemoradiotherapy (320). Peritoneal carcinomatosis and pelvic and abdominal tumors have also been successfully treated by hyperthermia in combination with radiotherapy (321, 322) as well as in combination with the platinum derivative Oxaliplatin (323–325) and also with Oxaliplatin + Irinotecan (326). A meta-analysis shows the feasibility of the method (47). Intraperitoneal chemoinfusion in the treatment of peritoneal carcinomatosis with malignant ascites had been treated (327), showing better results than the conventional therapy.

#### Superficial Tumors

Superficial tumors have been treated with hyperthermia with great success, mainly in combination with radiotherapy (246). The advantages can be seen well on the local control and local response-rate for melanoma (328–333), as well as for other superficial tumors (334–338).

#### Bladder

Hyperthermia can be applied for the urinary bladder (339). Survival periods are much longer (over 60% longer) when it’s applied with radiotherapy rather than with radiotherapy alone (340). It has also shown its efficacy in high-risk cases in combination with chemotherapy (341). Good results were achieved by hyperthermia plus Mitomycin-C in a randomized trial (342).

#### Soft Tissue

Hyperthermia has excellent results for soft tissue malignancies (343), especially sarcomas (89, 344, 345). The overall survival period in a long follow-up (over 10 years) was over 85% (346). Hyperthermia could be applied preoperatively (347, 348) and intraoperatively (349), and its whole-body application together with combined chemotherapies (Ifosfamide, Carboplatin, and Etoposide) is also published (350–353). Extensive research and clinical investigation was done on the topic by Issels and his group (347, 354–361). The research culminated in a large (*n* = 341) phase III randomized controlled trial (362) showing a 29.2% risk reduction (after 5.7 years median follow-up) in local progression or death when lesions were treated with loco-regional hyperthermia combined with surgery and radiotherapy. Some characteristic case reports (363) and a phase II clinical trial were also published with mEHT (364).

#### Experimental Applications

The combination of hyperthermia with gene therapy also looks promising, as shown by the successful combination of hyperthermia and heat-shock-protein (HSP) promoter-mediated gene therapy in advanced breast cancer patients (365). Hyperthermia improved the results of the HSP-promoter gene therapy by inducing local HSP production and by enhancing the local rate of release of HSPs from liposomes (366); this is also helpful for the double suicide gene transfer into prostate carcinoma cells (367). It was shown that this combination therapy was highly selective for mammary carcinoma cells. Also, heat-induced gene expression could be an excellent tool for targeted cancer gene therapy (368).

The combination of hyperthermia with hormone therapies is also a striking method. When applied to the prostate (369) all eight participants responded locally, and *in vitro*, quercetin and tamoxifen sensitize human melanoma cells to hyperthermia (370). The combination of hyperthermia with enzyme-therapy (371), PDT (372), gene therapy (373), immune- (374) and other supportive therapies (375) is also being investigated.

## Discussion

### The Challenges of Classical Hyperthermia

The history of success and the broad range of convincing results of oncological hyperthermia are not enough at the present moment to assure all medical experts of the feasibility of hyperthermia in oncology. Normally, a drug or method which has shown such clinical and research achievements and applicability would have been readily applied in therapeutic practice. Presently, less than 2% of cancer patients who would be indicated for hyperthermia, receive the therapy (376). Some health providers in clinical practices, decision makers, and professional insurers reject, or at least remain disinterested in considering hyperthermic therapy in oncology. Various factors contribute to the situation, including professional and emotional factors and a lack of knowledge of hyperthermia among doctors.

One of the most important factors contributing to the aversion of doctors to hyperthermia is the absence of an accepted definition of hyperthermia in oncology. Different websites, authors and industry leaders make use of different definitions and descriptions. Additionally, the technical challenges of heating up deep-seated tumors in a body create doubt. The number of devices available commercially is substantial, and in many instances manufacturers refer to the efficiency of their treatment based on phantom measurements, while relying on the clinical outcomes from other devices to prove efficacy. There is a concern that due to the variation in techniques and control mechanisms, not all of the techniques should be classified under one umbrella, and therefore the clinical data cannot be expected to be applicable to all of the techniques (377, 378). The broad assumption that all techniques have equal clinical outcomes is one consequence of the absence of a clear definition of oncological hyperthermia. This opens the door for techniques without sufficient clinical data to be marketed which, combined with a general inadequacy of knowledge about hyperthermia methods in oncology, supports the negative skepticism toward hyperthermia. The wide versatility of oncological hyperthermia applied to almost all types of tumors and at all stages of disease, with and without conventional therapies, fosters the “miracle universality” mind set of some clinicians which is naturally rejected by the professional community. The broad range of applications demands more and deeper research studies, and greater efforts to understand the impact of hyperthermia in malignant diseases. The responsibility for curing patients puts an enormous burden on the doctor, who will be hesitant to take responsibility for a therapy which is not completely understood or accepted. The relative simplicity and broad application of the heating techniques may cause uneasiness among professionals who expect the better defined protocols which they are familiar with for conventional therapies.

The difficulty determining the exact intratumoral temperature safely and accurately, along with the different effects observed at different temperatures, has resulted in significant debate amongst the hyperthermia community regarding the thermometry as a dosing and safety control parameter. Some techniques aim for temperatures in excess of 41°C, relying on the effects of higher temperatures on the DNA double strand break repair processes (379), while other techniques rely on moderate temperature increases of between 39 and 41°C (380), at which the improved perfusion enhances the effects of ionizing radiation on the tumor, or increases the drug delivery and reaction rate of chemotherapy in the tumor. Improved perfusion is seen in temperature increases as mild as 2°C (381) and at such mild temperatures, the immune modulating effects become more dominant (119). While attempts to define hyperthermia based on the temperature alone have been made (141), the variation in effects at different temperatures leaves a sense of incompletion in the definition of hyperthermia. This has allowed researchers to attribute the lack of positive results in some studies to inadequate heating or incorrect technique. For example, one study using a capacitive heating technique failed to show a benefit with the addition of hyperthermia to chemoradiotherapy for cervical cancer in a phase three study, despite measuring intratumoral temperatures in excess of 41°C (382). While another phase three randomized controlled study has shown significant benefit to the addition of mEHT to chemoradiotherapy for cervical cancer, despite not applying thermometry and likely only reaching temperature increases of 2°C (199). Analyses by Koresen et al. (380) have shown an association between temperature and outcome in their sample of patients treated with a radiative form of hyperthermia. It is our view that the problem lies with the dosing control measures, in other words the thermometry, and that going forward a change in the dosing method is required.

A potentially more complete definition would refrain from citing temperatures and rather describe applied energy to induce the desired effects, as defined by Szasz et al. (383): “Oncological hyperthermia is a method for killing malignant cells by controlled thermal effects, and has the potential to sensitize complementary therapies while avoiding the destruction of healthy cells.” A shift in the definition away from the historically temperature dependent version, could provide a solution to the challenge of treating deep tumors. However not all techniques are able to accurately determine the energy absorption and a substantial reworking of some models would be required to align with this new proposed dosing concept. This would be a big ask to change the dosing concept for a field which is only now gaining momentum, and is likely to meet with resistance from users who are comfortable with temperature as the primary dosing and safety parameter. When looking at the new paradigm, the only technique which currently fits into the proposed model is mEHT and while there are many advantages, including safety and ease of use, this technique is also not perfect.

The basic principles of classical hyperthermia have also been questioned. The ancient notion that heating the tumor would, by the increased temperature, kill the malignant cells, is an oversimplified explanation because it fails to consider the complex interactions of the tumor environment and the reactions of the human body when exposed to heat, such as the potential acceleration of transport of metabolites to the tumor, which may provide the tumor with extra support.

The absence of a common dose-concept of oncological hyperthermia presents doctors with seemingly vague conditions, which is another driving factor in the low degree of acceptance of the method (384). The tumor is extremely non-homogeneous by its nature. The physiological, electromagnetic and thermal parameters change dynamically, varying the developed temperature in various parts of the target. The most effective challenger of homogeneous heating is again blood flow. In local treatments where the cooling blood flow creates a dynamic heating instability, the thermal non-equivalence of the various parts of the tumor, both in the macro and micro ranges, defines the thermal maps. The theoretically applied dose of hyperthermic action is the CEM. It refers, at 43°C, to “x” percent of homogeneity (noted as the CEM43°CT_x_) (385). The reference measurement of the dose is provided by *in vitro* experiments on the necrosis of a cell-line at 43°C. The temperature was arbitrarily chosen, and based on a literature review at the time, the theoretical model that for every 1 degree decrease in temperature, a twofold increase in exposure time was required to induce necrosis, was developed (386). The reference-point has none of the physiological parameters or molecular alterations which could be important for complex processes.

A further challenge is that the CEM43°CT_x_ is too complicated and sometimes irrelevant (387): since it does not always correlate with clinical observations (348, 388). Its precise measurement is impossible in standard clinical conditions. The invasive temperature measurements are taken at discreet points, which is far too indefinite in such a non-homogeneous system, and the measurements cannot be taken as frequently as the treatment requires. In most publications, the medical staff measure the temperature intraluminally near the tumor. The lumen has much less of a “cooling” effect from blood flow than the tumor does, so the same energy absorption heats the intraluminal probe to a higher temperature, and there is no guarantee that the lumen is heated to the same energy flux or specific absorption rate (SAR) as the tumor. This is an indirect measurement of tumor-temperature and risks misleading the efficiency of the treatment.

Convincing experts in the clinical efficacy of the method is the next challenge. After a milestone publication by the Dutch Multi-Centre Alliance in The Lancet (185), expectations for hyperthermia were extremely high. The significant increase of the 4-year survival period for cervix carcinoma treated in combination with hyperthermia and radiotherapy, was a breakthrough. A repeated study however, did not show this difference (176), and the explanation (389) was the lack of a temperature reference point, a variation in methods, and thus a dose problem. A newer clinical study including a brachytherapy combination was also contraindicative (179). Importantly, the earlier clinical trials left unanswered questions. It was shown in 1996 (242) that radiotherapy with hyperthermia improved local control, compared to radiotherapy alone; however, the survival period was lower in the complete remission patients. This issue with the contradiction between the local control and survival period was also measured on superficial tumors in a clinical study (334). The earlier observed (164) toxicity problems reappeared.

Statistical reproducibility is in any case challenging. The majority of hyperthermia treatments are provided for patients where conventional therapies alone offer no further outcome benefits. This condition selects the advanced patient population, whose care would be largely palliative without hyperthermia. It is a great challenge to collect the proper cohorts of patients for statistical evaluation, because the pre-treatments and the conditions of patients have huge variations. Researchers avoiding this contra-selected population usually treat locally advanced cases without detectable distant metastases. This, however, decreases the enthusiasm of clinicians who are expecting solutions for complicated cases when the conventional treatment arsenal fails.

Most preclinical research is *in vitro* cell-line or *in vivo* small animal models. A vast proportion of the experimental research uses hyperthermia alone, while in the clinical practice, it is primarily used as a complementary and synergistic addition to conventional therapies. The monotherapy application of hyperthermia measures the direct thermal cytotoxic effects, but in clinics the complementary application of hyperthermia sensitizes and improves the efficacy of the curative actions of conventional therapies. In many model experiments, hyperthermia was applied with a water-bath and a homogeneous water-temperature was used as the basis of CEM43°CTx. Efforts are made rarely to conduct experiments similar to clinical applications, and which make use of various electromagnetic technical solutions. Another challenge related to the gap between research and clinical practice is the lack of human physiological effects included in the pre-clinical models. Furthermore, many animal experiments use artificially inoculated cancer cell-lines instead of naturally developed ones, which could differ from one another, especially when the tumor inoculation is not into the organ where the tumor would naturally grow. The systemic effects of local hyperthermia could face a challenge in small animal models, where the development of real metastases is relatively rare. Metastases are therefore modeled by injecting additional primary tumors into the animal.

The economic conditions are also not optimal for oncological hyperthermia. Most devices are expensive, have large installation and running costs, require costly labor and resources, and have much longer treatment times than do any other “gold standards of treatment.”

We have recognized the limitations and challenges of oncological hyperthermia. Recognizing the challenges is not enough to move forward, we need to formulate a method that can overcome the challenges and limitations. With this in mind, we propose a change in the paradigm of oncological hyperthermia.

### Proposal for the Change of Paradigm

The proposed change moves away from the strict control of the temperature as an outcome and instead defines the treatment based on the selective action of the absorbed energy, which induces a temperature elevation. In this model, isothermal temperature is not a requirement. The raised temperature is a condition and a tool for work, but the goal is the selective elimination of malignant cells. When considering the temperature increase, the period of increased temperature is temporary as the body naturally tries to eliminate temperature differences which normalize after the completion of the treatment. The temperature characteristically seeks homogeneity, and the traditional dose refers to this homogeneous temperature in the targeted area. But the heat naturally spreads by conduction and convection processes. The heterogeneity of the target and the blood flow through the vascular network in the target limits the homogeneity of temperature. The blood flow transfers the heat from the target to the rest of the body, and this further complicates the thermometry.

Another confounding factor is the transition from vasodilation to vasoconstriction beyond a certain temperature T_t_ in tumors. This is due to the growing intratumoral pressure and the walls of angiogenetic vessels, which lack the muscular structure to compensate for the increased pressure. Consequently, the value of T_t_ depends on the kind of tumor, and its stage. In theory, if the T_t_ is effectively achieved, then the vasocontracted tumor causes necrosis, which further blocks blood flow, reducing the cooling effects of the blood and makes it possible to achieve temperatures above the reference 43°C of CEM43°CT_x_ dose counting, which results in necrosis. However achieving and maintaining these temperatures are problematic. Additionally, the high gradient of blood flow in the outer region of the tumor (where active proliferation occurs) caused by the temperature growing over T_t_, increases the risk of dissemination and the advancement to distant the metastases.

Song observed the problem of T_t_ and opened a pioneering discussion on the topic (390). His proposal focused on mild, fever range hyperthermia. Song argues that the fever range is hot enough to improve perfusion and support complementary therapies depending on blood-transport, but not hot enough to promote the invasion and dissemination of the malignant cells. This idea closely represents the fine line between toxicity and therapeutic gain seen in other modalities.

The mild heating, when the temperatures do not exceed T_t_, stimulates general immune reactions. It was observed that the cytotoxicity of NK-cells sharply drops above 41°C (391, 392), and the activity of the immune-cells decreases. However, in the mild range an induced immune-effect is observed (393), even in the preoperative application (394). The suppression of the immune-activity by the high local tumor-temperature in LRHT is often neglected, with an assumption that new immune cells from the non-heated areas will be delivered.

The SAR characterizes the absorbed power in the target. The SAR and the temperature distribution do not correlate (395). The heating of unwanted materials in the target and the loss of energy due to the intensified blood flow and the massively forced surface cooling, modify the correlation between the SAR and temperature.

The new paradigm, described in detail in the following paragraphs, considers the regulation of blood flow, limiting it to avoid dissemination, but at the same time forming a synergy with complementary therapies, as well as inducing the appropriate distortion to the malignant cells. The goal is to ensure maximum absorption of the thermal energy (heat), within the malignant cells, without considerable heating of their environment and without heating the healthy surroundings at the same time. Heterogenic (selective) heating could be achieved by considering certain biophysical and physiological differences between cancer cells and their microenvironment from the non-malignant tissues in their vicinity (28). The clinically measured situation shows that a mild temperature gain together with a sufficient increase of blood flow, supports complementary therapies (28).

In the selection process, mEHT utilizes the fact that the cancer cells have increased proliferation, requiring appropriate nutrients, mainly glucose. The nutrients in the aqueous solution, such as the tissue electrolytes, represent higher ionic concentrations, which presents less resistivity to the electric current which is pushed through the target by the device. The current density in the tumor is selectively increased due to its lower impedance (396), and thus the lesion is selected. The microenvironment of the autonomous cancer cells differs from that of the healthy network. Healthy cells are connected, and their network enables the coordination of the cellular activities and mechanisms. Adding to the effects of the increased extracellular ions on the conductivity and permittivity of malignant tissue are the necrotic regions and the increased water concentration (although this does not result in the dilution of the electrolytes, it is only a change in volume which further decreases the conductivity and increases permittivity). Malignant tissues will therefore absorb more energy than the healthy tissues.

After the selection of the malignant tissue over the healthy tissue by mEHT (397), the energy absorption is focused in the malignant tissue. In a simulation on the nanoscopic effects on the cell membranes, Papp et al. demonstrated regions of focused energy absorption (398) which are believed to be the clusters of transmembrane proteins (rafts) on the membranes of cancer cells (399, 400). The malignant cells have relatively high raft density compared to the non-malignant neighbors (401), which appears to aid the selection further. This means that the energy-absorption could heat the membrane to at least 3°C higher than its surrounding extracellular electrolyte (402, 403). The full process from the temperature point of view shows the rise in temperature of the raft, representing the gradient generated by the mEHT action. In principle, the rafts will heat up the malignant cell and that heats the entire tumor. In this method the tumor temperature remains mildly increased, while the rafts have high energy-absorption. The goal is not to seek necrotic cell’s death, but to initiate an apoptotic signal transduction producing immunogenic cell’s death by causing damage to the cell membrane.

In order to target certain molecules within the tissue, the dispersion type at which the targeted molecules are affected must be determined and the frequency that is within that dispersion range must then be applied. In the case of mEHT, the target is the cellular membrane and the lipids and transmembrane proteins embedded in the membrane, and this is achieved by applying a frequency in the range of beta/delta-dispersion which targets these transmembrane proteins and lipids. The high beta/delta-dispersion causes an increased energy-absorption at the cell membrane of malignant cells which, as described previously, is due to the dipolar relaxation of membrane-bound and protein-bound water, and the rotation and relaxation of the of proteins and polar-side chains.

After this selection, the final step is the excitation by energy-absorption. Due to various physiological effects – various transport processes involving large (blood, lymph, nerve), small (junction, cadherins) and intracellular (cytoskeletons) transport mechanisms – mEHT applies a pattern recognizing and harmonizing fractal amplitude modulation (404) to keep the natural homeostatic control as effective as possible. The radiofrequency waves undergo amplitude modulation, at a frequency of 1/f (fractal range) which is out of the range of the malignant tissues and therefore causes increased and selective agitation of the malignant cells (404) while maintaining the natural homeostatic control as effective as possible.

**FIGURE 9:**
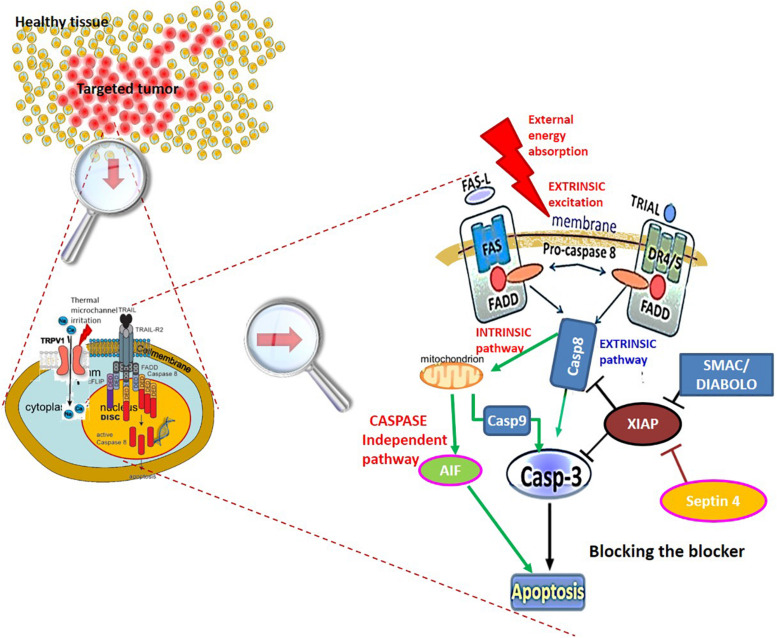
The five steps of the energy-selective mechanism causing apoptotic cell-destruction. The energy-absorption step (the 3rd one) is hyperthermic, proven by Arrhenius plot. The 4th step belongs to fractal-physiology. Extrinsic excitation and the apoptotic pathways to kill the cell with the mEHT method. Three variants of apoptotic signal pathway are used, and the blocker (XIAP) is blocked, preventing its action from limiting the signal transfer.

The mEHT process induces apoptosis by extrinsic excitation of several signal pathways (405–407). *In vitro*, the effects of mEHT as compared to conventional hyperthermia at the same temperature are significantly more profound (408). The strong synergy between the temperature and the field effects has been demonstrated (409). The electromagnetic excitation of the transmembrane proteins has an additional advantage: the promotion of apoptosis (410). The goal is the extrinsic excitation of the various apoptotic pathways (411), including the caspase dependent and independent processes (Figure 9). The extrinsic excitation targets the DR-5 death receptor in a complex with FADD and FAS molecules going through on both the path involving Caspase 8 and that involving Caspase 9 (Cas8 and Cas9) (408). It blocks the XIAP by Smac/Diabolo (412) and Septin4 (413), preventing its action against the signal transfer, and develops the apoptosis inducing factor (AIF) (408, 414) in a caspase-independent process.

The non-homogeneous induced heating in mEHT allows the application of a high precision hyperthermia (415), which is personalized (416). The well-focused energy absorption reduces the heating of non-malignant parts in the target, reducing the energy loss and increasing the efficacy of the treatment. The efficiency of the technique means that there is minimal loss of energy and most of the energy is deposited within the malignant tissue. This allows for the use of the forwarded, or incoming, energy as a measurement of the dose. The selected absorption of the power of the incident radiofrequency current allows us to introduce a simpler and biophysically accepted dose definition for the control of the medical process. This dose represents the same concept as is used in ionizing radiation, the Gray (Gy = J/kg). This dose is defined by absorbed energy, which is the product of the provided power with time. The absorbed power per unit mass is the SAR (W/kg). Connections between the SAR and the local temperature depend on physiological feedback mechanisms, a dependency which introduces a non-linearity to this relationship (417).

In mEHT, the selective heating allows for a good assumption of the amount of absorbed energy, and as a result the incident energy (J) can be used as the dose. An experiment in which artificial gold nanoparticles were added from suspension to the targeted volume and then heated with mEHT, demonstrates the selectivity of the technique (418). During the experiment, HepG2 cells were suspended and incubated with gold nano-particles and exposed to mEHT or a hat water bath. Using mEHT, no increase in temperature was detected and cell death rates were not increased. The authors concluded that the cell-incorporated gold nanoparticles reduced the cell selectivity and had a protective action to mEHT. The additional artificial particles in the naturally selected membrane rafts produce a higher quantity of energy-absorbing material while the cell-killing effect decreases. This is explained by the competing energy absorption between the (useful) membrane rafts and the nanoparticles incorporated into the cell, demonstrating that without the effects on the membrane rafts, the treatment does not have the desired effects.

The extracellular electrolyte of the selected malignant cells is heated more than the membrane-isolated cytosol. This is a result of the insulating effect of the cell membrane on the intracellular environment, which is described mathematically in an article by Szasz et al. (122). In the article, the tumor cells are described as collections of various electrolytes encapsulated by highly polarized thin films (various membranes), and the cells are suspended in an electrolyte rich solution (extracellular matrix). The thickness of the cell membrane ranges between 4 and 10 nm with a potential of between 70 to 90 mV and a capacity of 106F/m^2^. Additionally, the absorbed energy is at least two orders of magnitudes higher in the extracellular than in the cytosol (419, 420). The membrane acts as a barrier for electric field penetration into the cell, provided the applied frequency is not too high. The actions (excitation) are therefore primarily on the extracellular electrolytes and not on the intracellular electrolytes. These characteristics cause the membrane to act as a barrier for electric field penetration into the cell, provided the applied frequency is not too high. The actions (excitation) are therefore primarily on the extracellular electrolytes and not on the intracellular electrolytes.

## Future Prospects

The malignancy is a systemic disease. The goal of conventional LRHT is to eliminate the tumor, both with the highest achievement being declared as the reaching of complete remission. However, the appearance of metastases or relapses drastically limit the overall survival period of the patient. Chemotherapy or other systematically administered compounds (like check-point inhibitors, enzymes etc.) act systemically, with many variants in methods to activate personal immune actions against cancer. The challenge, however, is the highly adaptive hiding strategy of malignant cells, which protects them from the natural attacks of immune cells. An appropriate approach would therefore be to free the hidden genetic information of tumor cells and help the body to recognize and naturally kill the malignant cells. This could be achieved by stimulating immunogenic cell’s death, which results in the freeing of the genetic information from the tumor. This information may mature the dendritic cells (DCs). The matured DCs form CD4+ and CD8+ (helper and killer) T-cells with appropriate tumor-specific information, preparing them for tumor-specific immune attack (54). Immune preparation through antigen presentation could be achieved using an off-situ, extracorporeal, laboratory process as well.

**FIGURE 10:**
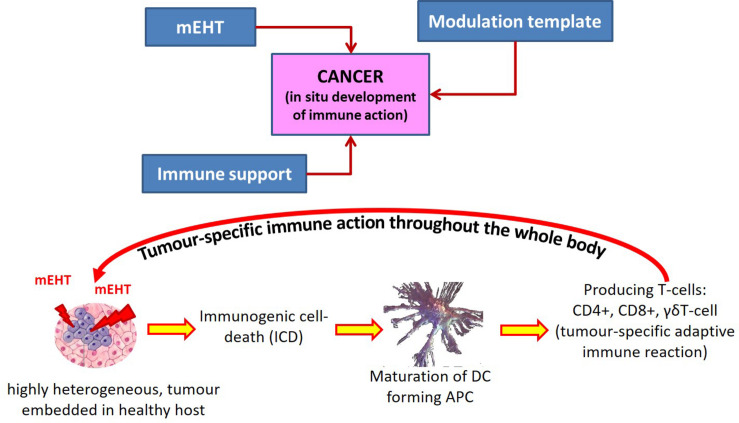
Immunogenic treatment with mEHT. The induced immunogenic cell-death presents the genetic information to DCs forming antigen presenting cells (APCs) and tumor-specific killer cells which are active all over the system. It is an *in situ*, real-time process.

There is also potential to include the mEHT treatments as a method of immune-modulation (421) to non-invasively stimulate the maturation of DCs. The stimulation of apoptosis during mEHT treatments could result in antigen presentation promoting the generation of CD4+ and CD8+ T-cells *in situ* inside the tumor (Figure 10).

A complication of mEHT immune treatment, however, occurs when the immune-status of the patient is weak. In this case, independent immune support is necessary through the offering of active DCs and macrophages. Antigen presenting cell (APC) production could occur when the immature immune-cells are available, but the high local temperature blocks their activity.

**FIGURE 11:**
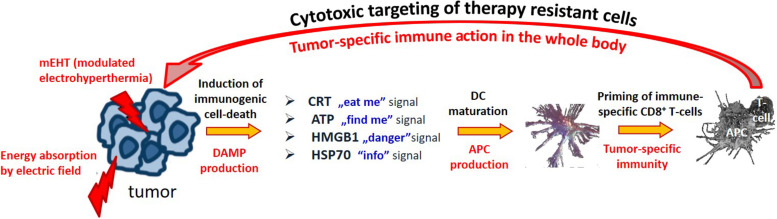
The molecular details of the immunogenic action of mEHT. The set of damage associated molecules (DAMP) has an important messenger role. The HSP is liberated from the cytosol, and becomes a game-changer: instead of protecting the tumor-cell, it helps to destroy it.

There is potential that the required cells are available outside of the treatment field (in the non-heated regions) and that these could be delivered to the tumor location after the heating is finished. Unfortunately, however this is not sufficient for *in situ* APC production. The immunogenic cell-death and subsequent APC formation is a complex process, requiring the production of a set of damage associated molecules (DAMPs) (422) which have to be available in a specific time sequence at the place of the formation of the APCs (Figure 11). The tumor-specific immune action could subsequently attack the malignant cells all over the body, irrespective of their distance from the treated primary lesion. The systemic abscopal effect could be enhanced by an immune-stimulating process (423), or by injected immature DCs (424), to improve APC formation. This immune action would work like a vaccination, with subsequent attacks by the same tumor being ineffective (425).

## Conclusion

Hyperthermia is a complex field, and it is further complicated by the variation in available techniques and the effects noted at different temperatures using the various techniques. The exact mechanisms of hyperthermia as a sensitizer are still not fully understood with debates on topics such as whether the inhibition of DNA repair is achieved at temperatures below 41°C, the impact of improved perfusion, and the timing of hyperthermia treatments, still underway (141, 380, 381). Many of the mechanisms of hyperthermia are still theoretical. For example the inhibition of DNA repair at temperatures in excess of 41°C has been demonstrated *in vitro*, but the required temperature *in vivo* cannot yet be proven due to thermometry challenges and difficulty obtaining tumors samples immediately before and after treatment in patients. The existence of membrane rafts is also not yet a widely accepted concept, although the membrane rafts fit with the model explaining the mechanisms behind mEHT. The effects of fractal range of modulation on the RF-waves in mEHT are not yet widely understood, however the hyperthermia community is recognizing the effects of the modulation and separate to the effects of heating (426). Pre-clinical and clinical studies demonstrated similar outcomes using lower temperatures with the addition of the amplitude modulation as compared to the same temperature with other heating techniques (199, 384, 412).

The immune effects at milder temperatures are intriguing, especially when considered parallel to the evolution of immunotherapies in oncology. The immunomodulating effects of hyperthermia are driving the research in hyperthermia in the direction of a more systemic and integrated modality in field of immuno-oncology. Immuno-oncology is an evolving field and the next few years are likely to shed more light on the interaction between the immune system and malignancies, and subsequently on drugs, ionizing radiation, and hyperthermia and the immune system. It is our view that this is the direction we can expect hyperthermia to move toward and we expect more research available on the immuno-modulating effects of hyperthermia, and the synergy between these effects and immunotherapies and radiotherapy.

The challenges with thermometry continue to plague the field with uncertainty, and the lack of thermometry in mEHT treatments causes a level of discomfort amongst clinicians who are still of the opinion that temperature is needed as a measure of treatment efficiency. Changing the dosing concept is an uncomfortable task for some clinicians and the discussions around the topic are continuing. When considering hyperthermia as a field, there is sufficient evidence from phase III randomized controlled trials to motivate the inclusion of hyperthermia in clinical protocols. However when considering each device and technique alone, the number of studies is limited. This leaves the clinician in a position where professional judgment and a good understanding of the field must guide the choice of technique, based on desired outcomes, resources, and feasibility. In the coming years we expect, as interest in the field grows, more studies on different technologies allowing for a comparison between effects and the development of protocols focusing on the techniques which have demonstrated improved outcomes.

The global market and healthcare is under pressure and technologies which are affordable and simple to use will become more important as healthcare systems around the world struggle under COVID-19 pandemic. The pandemic is likely to leave scars which could take years to overcome in some countries. We predict that there will be a shift toward the more simple and affordable heating techniques that can be easily taken up and integrated into the workflows at clinics. The COVID-19 pandemic has forced clinicians to think out of the box and to plan patient care to be as streamlined and effective as possible. Networking and the formation of multidisciplinary teams and improved collaboration between various medical fields and professionals has become more important. With this in mind, the need to apply a synergistic and multi-faceted approach to oncology protocols has been highlighted, and the uptake will likely be greater for technologies that can trigger systemic responses, as well as a local responses.

**FIGURE 12:**
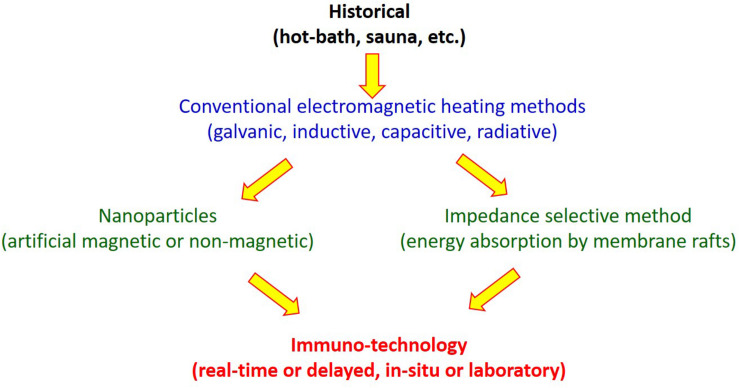
Development of the locoregional hyperthermia methods in oncology. The trend is in the direction of immune effects, demanding a new paradigm in hyperthermia.

It is our view that non-isothermal heating using mEHT has the potential to solve the old challenges and to usher in a new era of hyperthermia in oncology. The heterogenic molecular excitation demonstrated in preclinical studies on mEHT promotes tumor-specific immune-reactions which aligns with the shift toward immuno-oncology (Figure 12). Further understanding of the mechanisms involved in immuno-oncology will support the advancement of the field toward the direction of treating the patient as a whole system.

## Author Contributions

S-YL made broad literature research, categorized, and summarized it. GF helped the literature research in chemotherapy aspects. AMS evaluated the pathology aspects of the manuscript. GF and AMS wrote the manuscript. GS helped in search of the literature and its ordering as well as evaluation. AS investigated specific biophysical elements of the document and supervised the findings of the manuscript preparation project. CM added her wide practical expertise in thermo-radio-chemotherapy, evaluated the clinical aspects of the manuscript, and verified the whole manuscript and finalized it. All authors discussed the results and contributed to the final manuscript.

## Conflict of Interest

AS is Chief Scientific Officer of Oncotherm Kft. The remaining authors declare that the research was conducted in the absence of any commercial or financial relationships that could be construed as a potential conflict of interest.
